# Legume-Based Mobile Green Manure Can Increase Soil Nitrogen Availability and Yield of Organic Greenhouse Tomatoes

**DOI:** 10.3390/plants10112419

**Published:** 2021-11-09

**Authors:** Anastasios Gatsios, Georgia Ntatsi, Luisella Celi, Daniel Said-Pullicino, Anastasia Tampakaki, Dimitrios Savvas

**Affiliations:** 1 Laboratory of Vegetable Crops, Department of Crop Science, Agricultural University of Athens, 11855 Athens, Greece; gatsios@aua.gr (A.G.); ntatsi@aua.gr (G.N.); 2 Department of Agricultural, Forest and Food Sciences, University of Torino, 10095 Grugliasco, TO, Italy; luisella.celi@unito.it (L.C.); daniel.saidpullicino@unito.it (D.S.-P.); 3 Laboratory of Biological and Biotechnological Applications, Department of Agriculture, School of Agricultural Sciences, Hellenic Mediterranean University, 71004 Heraklion, Greece; atampakaki@hmu.gr

**Keywords:** *Solanum lycopersicum*, alfalfa pellet, faba bean, biological nitrogen fixation, organic tomato, soil nitrogen, rhizobia

## Abstract

Information about the availability of soil mineral nitrogen (N) in organic greenhouse tomatoes after the application of mobile green manure (MGM), and its impact on plant nutrient status and yield is scarce. Considering this knowledge gap, the effects of legume biomass from faba beans that are cultivated outdoors (FAB), or from feed-grade alfalfa pellets at two different doses (AAL = 330 g m^−2^; AAH = 660 g m^−2^) that were applied as MGM on the nutrition and yield of an organic greenhouse crop of tomatoes were evaluated. All of the MGM treatments increased the mineral N concentrations in the soil throughout the cropping period, and the total N concentration in tomato leaves when compared to the untreated control. FAB and AAH treatments had a stronger impact than AAL in all of the measured parameters. In addition, AAL, AAH, and FAB treatments increased the yield compared to the control by 19%, 33%, and 36%, respectively. The application of MGM, either as faba bean fresh biomass or as alfalfa dry pellets, in organic greenhouse tomatoes significantly increased the plant available soil N, improved N nutrition, and enhanced the fruit yield. However, the N mineralization rates after the MGM application were excessive during the initial cropping stages, followed by a marked decrease thereafter. This may impose an N deficiency during the late cropping period.

## 1. Introduction

Greenhouse tomato (*Solanum lycopersicum* L.) cultivation is an intensive cultivation system which requires a high nitrogen (N) input via fertilization to provide high yields [1,2]. According to Gianquinto et al. [3] the N requirements to produce 10 tons of tomato fruit range from 20 to 74 kg N, with an average of 35 kg.

Organic farming, on the other hand, is characterized by many restrictions and bottlenecks stemming from legislation or even from private production agreements [4]. The use of inorganic N in organic cropping is prohibited even if it is of mineral origin, according to European regulations [5,6]. Animal manure, which is an important source of organic N, is only allowed if it comes from organically raised or free-range animals. In addition, the European Nitrates Directive 91/676/EEC limits the application rate of manure to amounts of up to 170 kg N ha^−1^ yr^−1^. The application of the other main pillar of organic farming, namely compost, is quantitatively not restricted. However, compost has a low net N mineralization rate ranging between 5 and 25% of the total N content, depending on the C/N ratio, with the release of mineral N generally not exceeding 10% of the total N content [7,8]. Therefore, in organic greenhouse crops, it is difficult to meet the high N requirements of tomatoes, irrespective of growing in a long cropping cycle or in two shorter cropping cycles per year, as is common in southern Europe [9].

The contribution of legume green manure to N fertilization in organic farming through the biological N fixation (BNF) process has been well documented [10,11,12]. Specialized experiments that were carried out in both open fields [13,14,15] and greenhouses [16,17,18] have demonstrated that the incorporation of legume biomass into the soil can provide substantial amounts of plant available N for the following organic tomato crop. However, the cultivation and incorporation of green manure into organic greenhouses considerably reduces the available time for fruit production in the yearly cycle.

The method of cut-and-carry legume biomass and applying it as a biofertilizer (a practice frequently termed “mobile green manure” (MGM)), has been successfully tested in other crops [19,20,21]. However, it has been never tested in greenhouse tomatoes. According to this method, the legume is cultivated outside the greenhouse and, at the appropriate stage, is harvested, transported, and incorporated as green manure into the soil of the greenhouse field that is destined to accommodate the tomato crop. Thus, valuable space and time needed to grow legumes as green manure in the greenhouse is saved, while a high level of BNF can be maintained in the open field, which is more difficult in the fertile greenhouse soils [17,22,23]. A prerequisite for the application of this method is the availability of an open field in close proximity to the greenhouse, so as to reduce the transportation costs of the legume fresh biomass.

An alternative method of mobile green manuring is the application of alfalfa (*Medicago sativa* L.) pellets, which does not entail the availability of an open field for legume biomass production by the grower. Alfalfa pellets are available on the market as animal feed and have the advantage of easy storage and use when compared to fresh biomass. The beneficial effects of alfalfa pellets on the N nutrition of outdoor crops have been shown in other studies [19,24,25]. While the classic method of legume-based green manuring in the field offers only N and also provides some benefits with respect to phosphorus (P) availability, MGM enriches the soil with all of the macronutrients and trace elements that are necessary for plant growth. On the other hand, part of the benefit of legume crop rotation is due to the improvement of the soil properties and the reduction of plant pathogens [10,11,26]. This benefit is lost when MGM is applied.

Whatever the source and amount of total organic N that is supplied to the soil, the greatest challenge for organic crop nutrition is to synchronize the N mineralization rate with plant requirements [27,28,29]. Different plant species and varieties may exhibit different mineralization rates when incorporated into the soil as green manure [28]. Furthermore, fresh biomass may be characterized by higher decomposition rates when compared to dry biomass [28].

The determination of the mineralization rate in the laboratory has several limitations as the conditions are highly artificial and the estimate may be unrealistic [30]. On the other hand, the conditions in the field are dynamic and the estimation of the N mineralization rate is complicated by the absorption of the plants and other factors such as watering, especially with drip irrigation [30]. In addition, the needs of plants are affected by many factors such as propagation material, grafting, weather, soil conditions, etc. When the rate of N mineralization exceeds plant demand, the concentration of inorganic N in the soil increases and on the one hand there is a risk of N loss, mainly through leaching, on the other hand itcan also have adverse effects on plant growth. On the contrary, when the N mineralization rate is lower than the requirement of the plants, the concentration of inorganic N in the soil decreases and there is a risk of N deficiency in the plants.

Soil sampling and analysis for inorganic N (NH_4_, NO_3_) is a reliable tool for measuring the N availability; its main limitation is that it simply provides a snapshot in the time of sampling. Therefore, it is essential that sampling and analysis be repeated at regular intervals [31]. For this reason, this method is not particularly useful for routine analyses in agricultural production, but it is widely used for research purposes [32,33,34,35]. Sufficiency limits have been set by many researchers [34,36,37] for important crops, such as tomato. In addition, for all crops and conditions, the tendency of inorganic N concentration over time is a good indicator for assessing the level of N fertilization in relation to plant uptake [31,38]. Thus, in the case of organic farming, where N supply is based solely on mineralization, the tendency of inorganic N concentration over time is a useful tool for estimating the degree of synchronization of the N mineralization rate in relation to the plant N requirements.

Considering this background, the aim of the current study was to assess how green manure of legumes that are grown in an open field and transferred to an equal area in a greenhouse affects the total and inorganic N of the greenhouse soil, the plant N nutrition, and the yield of the organic greenhouse tomato. Furthermore, the impact of using alfalfa pellets at two different doses, low and high, on the above measured values was evaluated. A specific objective of the current study was to estimate whether the Ν mineralization rate of either the fresh bean biomass or the alfalfa pellets is synchronized with the tomato Ν requirements by measuring soil inorganic N at regular intervals.

## 2. Results

### 2.1. Legume Biomass, BNF and Soil N Supply

The amount of organic N that was applied via MGM was 100, 200, and 150 kg N ha^−1^ in the AAL, AAH, and FAB treatments, respectively (Table 1). In addition, the C/N ratio was 12.4 in alfalfa pellets treatment and 10.3 in the faba (*Vicia faba* L.) bean treatment. The N derived that was from the atmosphere (Ndfa) in the faba bean tissue was 83.2% and thus the biologically fixed N amounted to 125 kg N ha^−1^.

### 2.2. Soil Measurements

**Figure 1:**
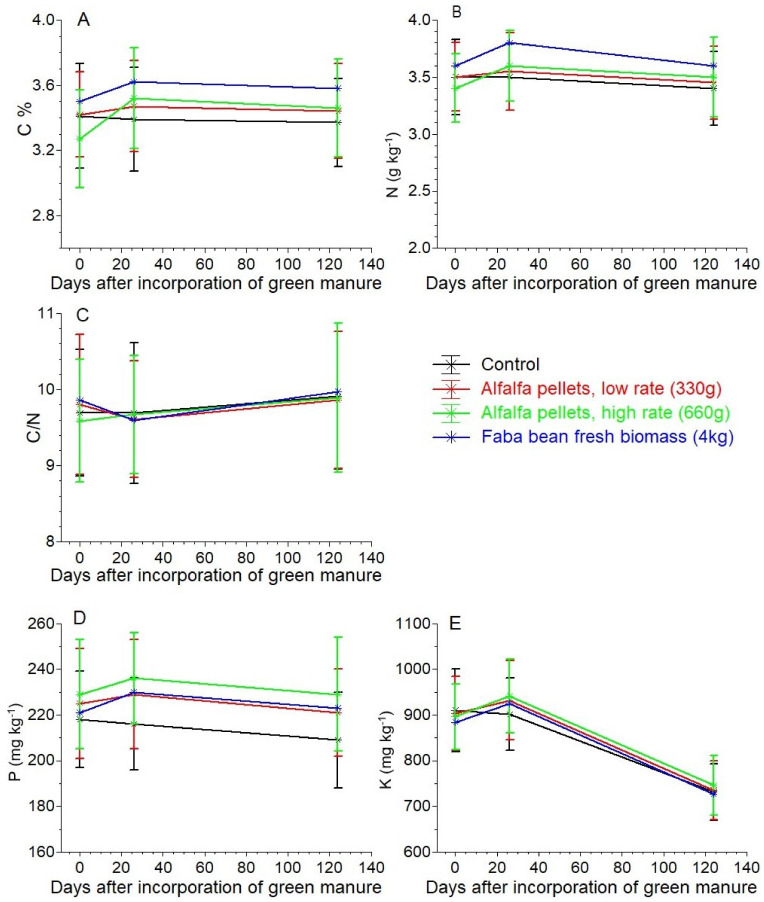
Organic C (**A**), total-N (**B**), C/N ratio (**C**), available P (**D**) and K (**E**), in dried soil before legume biomass incorporation (D 0), after tomato transplanting (D 26) and at tomato crop termination (D 124). The vertical bars represent ± standard errors of means.

**Figure 2:**
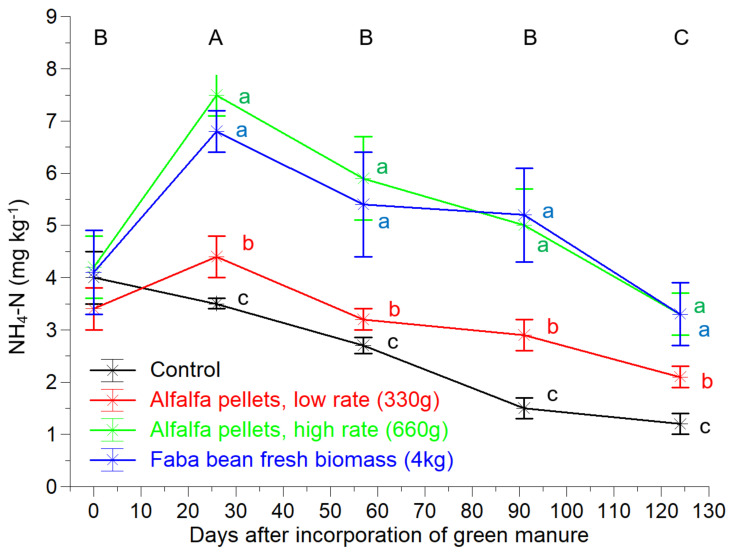
Soil NH_4_-N concentrations in an organic greenhouse crop of tomatoes as influenced by different treatments of mobile green manure. The vertical bars represent ± standard errors of means. The different lowercase and capital letters indicate significant differences between the treatments and sampling dates, respectively, according to the Duncan’s multiple range test (*p* < 0.05).

The different forms and rates of MGM did not significantly influence the soil organic carbon © and total N concentrations (Figure 1A,B). Similar to the total N and C, the concentrations of the available P and potassium (K) in the soil were not significantly affected by any treatment (Figure 1D,E). The soil ΝH_4_-N concentration was constantly low across all of the sampling dates and all of the treatments, with values below 8 mg kg^−1^ (Figure 2). The highest soil ΝH_4_-N concentrations were measured in the AAH and FAB treatments, without significant differences between them, followed by the AAL, while the lowest levels were found in the control. The ΝH_4_-N concentrations in the control were constantly declining, while in the other 3 treatments a peak was observed after incorporation of the organic matter, followed by a declining trend thereafter.

**Figure 3:**
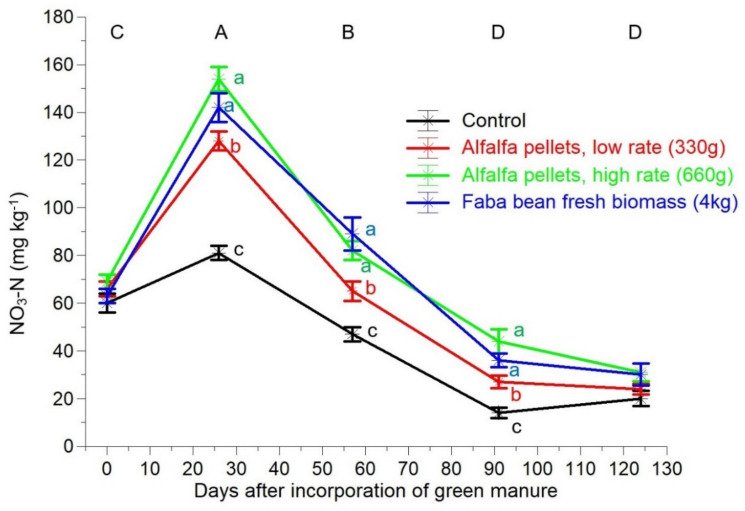
Soil NO_3_-N concentrations in an organic greenhouse crop of tomatoes as influenced by different treatments of mobile green manure. The vertical bars represent ± standard errors of means. The different lowercase and capital letters indicate significant differences between treatments and sampling dates, respectively, according to the Duncan’s multiple range test (*p* < 0.05).

The ΝO_3_-Ν concentration in all of the treatments just before the incorporation ranged between 60 and 69 mg kg^−1^ with no significant differences between the treatments (Figure 3). Subsequently, there was a peak in all of the treatments 25 days after the incorporation of the green manure (DAIGM), which was followed by a sharp decrease thereafter. In all of the post-incorporation samplings, the ΝO_3_-Ν concentrations in the AAH and FAB treatments were similar and significantly higher than in the AAL treatment, while in the control it was appreciably lower than in all three of the green manure treatments.

### 2.3. Tomato Growth, Yield and Yield Components

In the early stages of cultivation, the tomato plants showed signs of strong vegetative growth. Subsequently, the growth became normal, first in the control and then in the three treatments with MGM, but all of the treatments showed symptoms of N deficiency two and a half months after transplantation. The tomato fruit yield was similar in the AAH and FAB treatments and significantly higher than in the other two treatments, while the AAL treatment rendered a significantly higher yield than the control (Table 2). The differences in the tomato yield were due to both number of fruits per plant and the average fruit weight.

### 2.4. Tomato Tissue Analysis and N Partitioning to Fruit

The N concentration in the leaves was appreciably higher in the FAB and AAH treatments compared to the other two treatments, without significant differences between them, while the concentration of leaf organic N in the AAL treatment was significantly higher than in the control (Table 3). In contrast, the leaf P and K concentrations were not affected by any treatment.

Unlike the leaves, the tomato fruit exhibited similar N levels in all of the treatments (Table 4), while the fruit P and K concentrations were also not influenced by fertilizer treatments tested in this study. However, due to the higher yield, the organic N that was removed from the field due to the harvesting of fruit was significantly higher in the FAB and AAH compared to the other two treatments.

## 3. Discussion

### 3.1. Faba Bean Aboveground Biomass, N Accumulation and BNF

The Ndfa percentage in faba beans that were grown in an open field was high (83.2%), thereby corroborating previous reports which showed that this legume is characterized by a high N_2_ fixing ability [1,11,39]. A high Ndfa is more difficult to achieve in greenhouses as the symbiotic N fixation of legumes is appreciably reduced when substantial inorganic N is available in the soil [17,22,23]. Therefore, when a legume is cultivated as green manure in fertile greenhouse soils, the net N supply may be low due to decreased BNF. In the current study, the biologically fixed N was 125 kg N ha^−1^, which was somewhat lower than in other studies [11,40]. Considering that the N concentration in the tissues was in line with expected contents (3.93%), the lower BNF is ascribed to the rather low amount (4 kg m^−2^) of faba bean aboveground biomass that is produced [23]. The relatively low biomass production in the faba bean crop is a result of early harvesting, which took place before anthesis. However, this is inevitable because the establishment of the subsequent tomato crop cannot be delayed beyond mid-February, as dictated by local market requirements.

Although the belowground biomass of faba beans were not measured in the current study, it is estimated that the N deposited to the roots accounts for more than 20% of the total N [11,39,41]. Given the high Ndfa, we can conclude that the balance between N input through BNF and N output through the aboveground biomass harvest was positive in the FAB treatment with respect to N. Therefore, the cultivation of faba beans is sustainable in terms of N, while P and K must be maintained at the desired levels by providing organic or inorganic fertilizers of natural origin in the open field. On the other hand, the application of legume biomass to the greenhouse soil provides not only N, but all of the necessary macronutrients and trace elements for plant growth.

### 3.2. Soil Nutrient Availability

The greenhouse soil contained high organic matter (3.27–3.50% C) and total N (0.34–0.36%). Therefore, the impact of the three fertilizer treatments on soil organic C and total N was insignificant. As reported by Peoples et al. [42], the influence of legume green manuring on the total N soil is not always easy to detect but the effect on the inorganic N level is much more consistent. Also, according to Sainju et al. [35] repetitive applications of green manure are necessary to impose significant differences in organic soil C and N as a cumulative effect. In addition, the soil P and K concentrations before the incorporation of the organic green manure were already sufficient for tomato crops [3,36,43] and thus an effect of the treatments could not be detected. (Figure 1D,E)

Although the NH_4_-N concentrations were significantly different, their levels were relatively in all of the treatments. This is attributed to the high nitrification rate of NH_4_-N that characterizes nonacidic topsoils with sufficient aeration and microbial activity [44,45,46].

Prior to incorporation of green manure, the NO_3_-N concentrations were high in all of the treatments, reflecting the high levels of total N and the low C/N ratio (<10, Figure 1C) in the soil [45,47]. The increase that occurred in the next sampling time (26 DAIGM) in all of the treatments, even in the untreated control, is attributed to the absence of N uptake as the tomato plants were established 20 DAIGM. The sharper increase in the green manure treatments is attributed to the high rate of mineralization that occurs in the first weeks after the incorporation of legume biomass [33,48,49]. However, the concentrations in these three treatments exceeded the suitable levels of NO_3_-N, which are 50 to 100 mg kg^−1^, according to van Eysinga [37] and Sainju et al. [36]. Subsequently (57 DAIGM), the soil NO_3_-N levels in the treatments of MGM were within the optimal range, whereas in the control they were low [34,50,51]. In the last two samplings, the differences in NO_3_-N concentrations between treatments persisted but fluctuated below the sufficient NO_3_-N level for tomato N nutrition. Finally, while 50 kg ha^−1^ (33%) more N was added in the AAH treatment compared to the FAB treatment, their effect on the soil mineral N was consistently similar at all of the sampling dates.

### 3.3. Tomato Growth and Yield

During the first six weeks after transplanting, when mineral N and especially ΝO_3_-Ν levels were excessively high, the tomato plants developed curled thick leaves and a thick stem at the top in all of the treatments except the control. This appearance was characteristic of vegetative growth that was too vigorous, which was attributed to excessively high N levels in the root zone [52]. Around the time of the third soil sampling date (57 DAIGM), the plants had returned to normal growth and set fruit abundantly in the first four clusters. However, this growth phase was followed by the gradual development of typical N deficiency symptoms, mainly manifested as thin stems and light green leaves [36,52], which appeared first in the control and about three weeks later also in the MGM treatments. The visual diagnosis of N deficiency was corroborated by leaf analysis that was performed 75 days after transplanting. Indeed, at that date the leaf N concentrations in all of the treatments were lower than 30 mg g^−1^, which is the minimum threshold for adequate N nutrition in tomatoes [3,36]. The addition of extra organic N to all of the treatments was performed after this stage and therefore did not affect the N nutrition and the identified N deficiency. Nevertheless, the leaf P and K concentrations in all of the treatments were within the optimal range [3,36], as the soil was very well supplied with these macronutrients. According to the literature [32,53,54] the most limiting factor for yield in organic farming is the soil N level. The present study corroborates this consideration, as the level of tomato fruit yield was commensurate with the concentration of mineral soil N. Nevertheless, the MGM provided clear benefits to organic greenhouse tomatoes, as it increased the soil mineral N, and, concomitantly, the fruit yield when compared to the control, while it delayed the occurrence of N deficiency symptoms.

As reported by many researchers [27,28,29], the synchronization of N release through decomposition of organic matter with plant N requirements is a major challenge in organic crops. The current study showed that legume biomass that is applied as green manure in greenhouse tomatoes cannot successfully address this challenge as it is rapidly decomposed during the first four to five weeks after its incorporation into the soil, in agreement with previous studies [15,16,45]. The rapid decomposition of legumes that were applied as green manure is ascribed to the low C/N ratio in the legume biomass [28]. To increase the C/N ratio and decrease the mineralization rate of the biomass that is incorporated to the soil as green manure, faba beans or any other legume might be intercropped with a grass (e.g., rye) [49,55,56]. Similarly, instead of applying feed-grade alfalfa pellets, a mixture of alfalfa and grass, compost, or other organic material may be manufactured with the same aim of increasing the C/N ratio in the biomass that is used for fertilization of organic greenhouse tomatoes.

According to Benincasa et al. [55] and Sorensen and Thorup-Kristensen [57], as the ontogeny of the plants progresses, the nutrient content decreases due to the dilution effect. Thus, the C/N ratio in alfalfa biomass can increase from 10 at early harvest to 20 at late harvest [57], and in faba bean from 11 to 16, respectively [21]. Thus, the C/N ratio can also be raised by properly manipulating the developmental stage at which the legume is incorporated into the soil. This strategy contradicts the suggestion of Sorensen and Grevsen [21] to apply green manure with a low C/N ratio, but this suggestion may be only valid to open-field crops in temperate climates. Under greenhouse conditions, high soil temperature and the optimal humidity conditions favor a rapid decomposition of organic matter, while the duration of greenhouse tomato cultivation is relatively long. Thus, under greenhouse conditions, it seems advisable to apply biomass with a higher C/N ratio as green manure to avoid excessive N mineralization rates immediately after its incorporation into the soil followed by poor mineral N release thereafter.

In the current study, the application rate of alfalfa pellets had a significant impact on both the soil N levels and the fruit yield. Thus, any increase of the C/N ratio in the biomass that was applied as green manure in organic greenhouse tomatoes should be accompanied by a commensurate increase in the total applied biomass to maintain a high N supply level to the crop. Nevertheless, although an increase of the C/N ratio in the biomass that is incorporated into the soil as green manure may delay the occurrence of N deficiency in greenhouse tomatoes, presumably it cannot prevent it completely due to their high N requirements and the long cropping period. Thus, the application of water-soluble organic N fertilizers that are permitted in organic farming through fertigation after a particular cropping period, or alternatively, the N application as top dressing (feather meal, brewery waste, etc.) may be the only sustainable cropping practice to maintain the yield potential of tomatoes that are grown organically in greenhouses [4].

In this research, the removal of N from the soil through the vegetative plant components had been recouped in advance as the residues of the previous tomato crop had been incorporated into the soil in all of the treatments, following common practices in organic farming [2,29]. Leaching of NO_3_-N in greenhouses is minimal when a proper irrigation scheduling through drip irrigation is applied [3], while N losses due to denitrification as N_2_O emissions were low (data are not shown). Thus, in the current study, the N removal through fruit harvesting can be considered a good estimate of the total N removal from the soil. The fruit N concentrations were low compared with those that were reported in the relevant literature for conventional tomatoes [36,58]; in organic farming, the fruit N concentration is usually lower than in conventional crops [14,58,59]. Accordingly, the N removal through the harvested fruit, which ranged from 7.6 to 11.2 g m^−2^, was also low. These N amounts are similar to those that were provided by the AAL treatment and lower than those that were provided by the AAH and FAB treatments. However, the NO_3_-N and NH_4_-N levels were substantially lower at the end of the cultivation period compared to day zero, i.e., before the incorporation of the MGM into the soil. This gradual decrease of the soil mineral N in all of the treatments that were below the levels that were measured before the incorporation of the organic biomass indicates that, after a particular cropping stage, the plants were acquiring part of their N requirements from the soil reserves. This indicates that the mineralization rates after the initial peak decreased to levels that were incapable of covering the plant N requirements, since, as stated above, the total amount of N that was included in the organic matter was sufficient to cover the N removal for the fruit in all of the treatments of MGM.

The highest tomato yields were recorded in the AAH and FAB treatments, 13 kg m^−2^ and 13.3 kg m^−2^, respectively. These yields are comparable to the average yields of conventional tomato cultivation in the same region and for the same growing season, according to data from the Directorate of Rural Economy and Veterinary of the Preveza Regional Unit (T. Stavropoulou, personal communication, 27 September 2021). According to the data of the above local agricultural service, the prices of organic tomato fruits are at least 30% higher than those that are achieved by conventionally produced tomatoes throughout the year. Therefore, the income from organic tomato cultivation in the greenhouse is estimated to be significantly higher than from the conventional greenhouse tomato crops.

Finally, the FAΒ application is economically more advantageous as a lower addition of organic N had the same effect on N nutrition and yield of tomatoes as AAH, while the latter is additionally charged with the costs of drying, transport, and marketing. In addition, the area that was cultivated with faba beans, or any other legumes that are used to apply mobile green manuring, can be extended in relation to the greenhouse area. Finally, the FAB treatment can be further improved by extending the growing season of the legumes to enhance the net amount of N that is provided to the tomato crop, as well as by intercropping with grasses, thereby increasing the C/N ratio to obtain a more even course of N mineralization.

## 4. Materials and Methods

### 4.1. Plant Material, Growth Conditions, and Treatments

**Figure 4:**
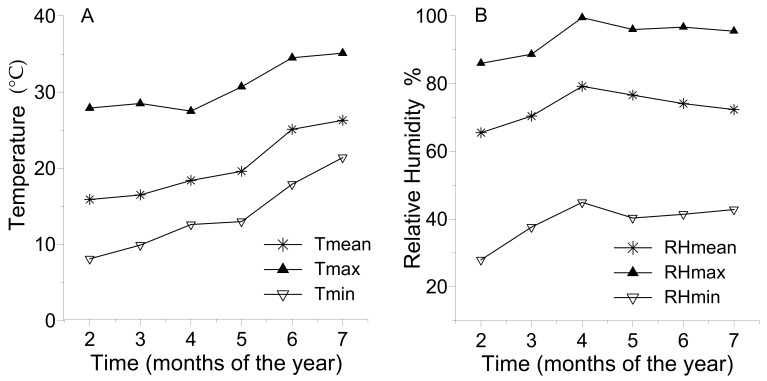
Monthly averages of mean, maximum, and minimum daily temperatures (Tmean, Tmax, and Tmin, respectively, Panel (**A**)) and relative humidity (RHmean, RHmax, and RHmin, respectively, Panel (**B**)) inside the experimental greenhouse during 2019.

In a greenhouse experiment, organic tomatoes were grown from January 2019 to July 2019. The experiment was carried out in Preveza, northwestern Greece (38°59′29.2″ N; 20°45′36.1″ E, 5 m a.s.l.) in a commercial arch type greenhouse (NNE–SSW oriented) with vertical sidewalls, covered by low-density polyethylene film. The size of each plot was 3.75 m × 5.00 m (i.e., 18.75 m^2^). The soil type was a sandy loam with a neutral pH (7.3 measured in water extract). The monthly average temperatures (mean, maximum, minimum) and relative humidity (%) are presented in Figure 4.

A total of four different treatments with four replicates per treatment were established in February 2019. The preceding crop were also tomatoes in all of the treatments and 5 days before its establishment (on 7 August 2018) farm-yard manure originating from free-range cattle farming, containing 0.34% N, 0.15% P, and 0.48% K, had been applied. The application rate was set to 50 t ha^−1^ representing an input of 170 kg N ha^−1^, which is the maximum allowed level per year according to Commission Regulation (EC) 889/2005 [5].

In Treatment 1 (CON), which is considered the control, no legume biomass was incorporated into the soil before the establishment of the tomato crop. In Treatments 2 (AAL) and 3 (AAH), alfalfa pellets were applied at two doses, specifically 330 g m^−2^ and 660 g m^−2^, respectively. In Treatment 4 (FAB), faba bean fresh aboveground biomass that was grown in an open field next to the experimental greenhouse was incorporated into the soil after the quantification of the total aboveground dry mass and the tissue N concentration.

The outdoor cultivation of the faba bean area that was used in Treatment 4 occupied a similar area to the indoor cultivated tomatoes in the corresponding treatment (75 m^2^). The seeds of the faba beans that were cultivated outdoors for the needs of Treatment 4 were inoculated with *Rhizobium* sp. VFLE1 [60] and sown on the 16 October 2018. The aboveground biomass of the faba beans was harvested on 31 January 2019, i.e., 3.5 months after sowing.

The legume biomass (alfalfa pellets or faba bean) was incorporated into the soil on 1 February 2019 in Treatments 2, 3, and 4, and the tomato crop was established 20 days later in all of the treatments. The commercial tomato hybrid ‘Nissos F1’ (Hazera Seeds Ltd. Tel Aviv, Israel) that was grafted onto the commercial rootstock ‘Maxifort F1’ (*Solanum lycopersicum* × *Solanum habrochaites,* De Ruiter Seeds, Bergschenhoek, The Netherlands) was used as planting material. The grafted tomato seedlings were transplanted to the greenhouse at a density of 2.13 plants/m^2^. All of the plants were pruned to a single stem and were drip-irrigated. During the tomato cultivation, additional N (13 kg N ha^−1^, added in three separate doses with equal amounts at each dose) was provided to all of the treatments through the irrigation water using an organic N fertilizer that was certified for organic farming. More specifically, the extra N was provided to all treatments at 76, 85, and 98 days after planting.

### 4.2. Growth, Mineral Analysis, and N Fixation by Legumes

The aboveground biomass of the faba beans within the 0.5 m × 0.5 m quadrants was collected in quadruplicate and the dry mass determined after the tissue samples were oven-dried at 65 °C to a constant weight. The dry plant tissue was powdered using a ball mill, was passed through a sieve (0.5 mm), and was analysed for organic C and total N using an elemental analyzer (Unicube, Elementar Analysensysteme GmbH, Hanau, Germany). The total P concentrations in the plant tissues were determined by ashing at 550 °C for 8 h, dissolving the soluble salts in 4 M HCl, and quantifying P in the extracts using a spectrophotometer (U-2000, Hitachi, Tokyo, Japan) following the molybdate blue method [61]. Potassium was determined in the same aqueous extract using a flame photometer (Sherwood Model 410, Cambridge, UK).

The %Ndfa in the aboveground biomass of the faba beans was determined by applying a method that was based on the natural abundance of ^15^N in plant tissues relative to the air [62,63]. The grass weed *Digitaria sanguinalis* (L.) was used as non-N_2_-fixing reference plant in this assay. Subsequently, the obtained %Ndfa values were used in combination with the total dry biomass of the faba beans to determine the total amounts of biologically-fixed N_2_ by the faba beans per cultivated area unit (BNF, kg ha^−1^). Detailed description of the methodology that were applied for the determination of %Ndfa was provided by Gatsios et al. [17].

### 4.3. Tomato Tissue Sampling and Mineral Analysis

To determine the nutritional status of the tomatoes, samples of youngest fully expanded leaves were collected from 5 plants in each plot 75 days after planting, according to Gianquinto et al. [3]. Sampling was performed after the fruits started ripening and before the top of the stem was removed so that the plant high N requirements at this stage would reveal a possible N deficiency and significant differences between treatments. The leaves were washed with distilled water, chopped, and oven-dried at 65 °C until they reached a constant weight, powdered using a ball mill, and passed through a sieve with mesh size 0.5 mm. Subsequently, the total N, P, and K concentrations were measured as described by Gatsios et al. [17]. Furthermore, ripe tomato fruits that were collected from the second cluster of 5 plants in each plot were analyzed according to the same procedures to determine their total N, P, and K concentrations.

### 4.4. Soil Analysis

Soil samples were collected from the central square of each plot (dimensions 2 m × 2.5 m). In each plot, 5 soil cores, each weighing about 400 g, were collected from the root zone of 5 plants at a depth of 0–20 cm. The sampling was repeated at regular intervals to determine the availability of soil N at successive crop stages and to assess the tendency of N concentration during tomato cultivation. The samples were prepared according to Miller et al. [64] and analyzed to determine the total-N, NO_3_-N, NH_4_-N, and plant-available P and K concentrations according to the methods described previously [17].

### 4.5. Tomato Yield and N Fruit Uptake

The fruit yield was assessed by harvesting all of the ripe tomatoes every 3 days from 10 plants of the plot center and recording their total weight and number per plant. The nitrogen partitioning to the fruit was calculated for each experimental plot by multiplying the fruit N content by the dry matter percentage and the yield.

### 4.6. Statistical Analysis

The experiment was set as a randomized complete block design with 4 treatments and 4 replicates per treatment. A total of 16 experimental plots were divided into 4 blocks and in each block a replication from each of the 4 treatments was carried out. The data were statistically analyzed by applying an ANOVA (STATISTICA, version 12.0 for Windows, StatSoft Inc., Tulsa, OK, USA). The means were separated by applying the Duncan’s multiple range test when an ANOVA was significant at *p* < 0.05. The data are presented in graphs and tables as means ± SE of the four replicates. Linearity, normality, homogeneity of variance, and influential values in the residuals were assessed for every statistical model that was applied.

## 5. Conclusions

The application of MGM, either as fresh faba bean biomass or as alfalfa pellets, significantly increased the available plant N in the soil and concomitantly the plant N nutrition and the fruit yield in organic tomato crops that were cultivated in greenhouses. The benefit to the tomato crop increases with the application rate of alfalfa pellets, but the addition of N through fresh faba bean biomass seems to be more effective and economically advantageous.

However, N mineralization rates did not seem to be synchronized with the crop N demand, and this resulted in an unbalanced N supply that may have imposed N excess in the early stage and N deficiency at a later cropping stage. With the expansion of the faba bean cultivated area and appropriate manipulation, the amount of N and the ratio of C/N can be increased, to mitigate this imbalance in the N nutrition of the tomato crop. Nevertheless, supplementary organic N supply through fertigation or top dressing seems to be necessary in greenhouses when a tomato is cultivated organically to maintain the productivity level of the crop for a longer period.

## Figures and Tables

**Table 1:** Dry biomass, tissue N and C concentrations, C/N ratio and total N input provided via mobile green manure in each treatment.

Treatment Short Name	Treatment Description	Dry Biomass g m^−2^	N %	C %	C/N Ratio	N Input g m^−2^
CON	Control	-	-	-		-
AAL	Alfalfa pellets, low rate (330 g m^−2^)	297	3.37	41.8	12.4	10
AAH	Alfalfa pellets, high rate (660 g m^−2^)	594	3.37	41.8	12.4	20
FAB	Faba bean fresh biomass (4 kg m^−2^)	382	3.93	40.4	10.3	15

**Table 2:** The impact of different organic fertilization treatments on organic greenhouse tomato yield components.

Treatment	Yield kg m^−2^	Fruit Number per Plant	Mean Fruit Weight
Control	9.8 c	20.2 b	228 b
Alfalfa pellets, low rate (330 g m^−2^)	11.7 b	23.7 a	231 b
Alfalfa pellets, high rate (660 g m^−2^)	13.0 a	24.0 a	254 a
Faba bean fresh biomass (4 kg m^−2^)	13.3 a	25.7 a	244 a
Significance of differences	***	**	**

Means (*n* = 4) followed by different letters within each column indicate significant differences according to the Duncan’s multiple range test (*p* < 0.05); **, *** significant *p* < 0.01, and *p* < 0.001, respectively; ns = not significant.

**Table 3:** The impact of different organic fertilization treatments on leaf N, P, and K dry matter concentrations in organic greenhouse tomato.

Treatment	N mg g^−1^	P mg g^−1^	K mg g^−1^
Control	23.9 c	2.78	78
Alfalfa pellets, low rate (330 g m^−2^)	26.6 b	2.80	81
Alfalfa pellets, high rate (660 g m^−2^)	28.1 a	2.99	86
Faba bean fresh biomass (4 kg m^−2^)	28.8 a	2.90	84
Significance of differences	***	ns	ns

Means (*n* = 4) followed by different letters within each column indicate significant differences according to the Duncan’s multiple range test (*p* < 0.05); *** significant at *p* < 0.001; ns = not significant.

**Table 4:** The impact of different organic fertilization treatments on fruit N, P, and K dry matter concentrations and N removal by fruit harvesting in organic greenhouse tomato.

Treatment	N mg g^−1^	P mg g^−1^	K mg g^−1^	N Removal through Fruit g m^−2^
Control	15.2	2.81	81	7.6 c
Alfalfa pellets, low rate (330 g m^−2^)	15.5	2.61	84	8.9 b
Alfalfa pellets, high rate (660 g m^−2^)	16.2	2.84	81	10.7 a
Faba bean fresh biomass (4 kg m^−2^)	16.8	2.65	75	11.2 a
Significance of differences	ns	ns	ns	***

Means (*n* = 4) followed by different letters within each column indicate significant differences according to the Duncan’s multiple range test (*p* < 0.05); *** significant at *p* < 0.001; ns = not significant.

## Data Availability

Data is contained within the article.
